# Notes and News

**Published:** 1905-04-08

**Authors:** 


### Notes and News.

The King has sent a donation of 100 guineas to the West Norfolk and Lynn Hospital, and has increased his annual subscription to 30 guineas.

The Viscount Hampden has accepted a seat on the board of management of the National Hospital for the Paralysed and Epileptic.

The Metropolitan Asylums Board have consented to receive a deputation from the Metropolitan Branch of the Incorporated Society of Medical Officers of Health to urge the need of the Board to undertake to provide accommodation for metropolitan consumptives, and to become the sanatorium authority for the metropolis.

The report that the Mission to Deep Sea Fishermen claimed £14,000 damages against the Russian Government is not a fact. The Mission claimed £5,042 for damages to the *Alpha* and received £3,985. It appears that the actual cost of repairs, etc., exceeded the award by £79. The claim for four months' detention of the vessel from her proper work and depreciation was ignored.

At an inquest at Farnborough a system of treatment by post played a very prominent part. A resident at Farnborough, aged 68, placed himself in the hands of the Weidhaas Hygienic Institution, Burgess Hill. It appears that the institute claimed to cure complaints by "Psychic Therapy." The treatment, in practice, appeared to consist largely of the administration of a herbal concoction called "Star Tea Tonic." Before treatment by post the patient was required to fill in a most elaborate form of inquiry as to his symptoms. The actual cause of death was certified to be heart disease, and medical evidence went to show that there was no reason to show that the "treatment by post" had been harmful, but it was pointed out that had a medical man been consulted the serious condition of the deceased's health would have been at once ascertained. Treatment and prescribing by unqualified persons was unfavourably commented on.

Lord Derby has presented £1,000 to the Royal Sea Bathing Infirmary at Margate.

Mr. Alfred Beit has contributed £4,000 to the Guy's Hospital Appeal.

The late Sir Edward Scott has bequeathed £5,000 to the Walsall Hospital.

The late Miss Thomasina Fawsett has bequeathed £2,000 each to the Royal Hospital for Incurables and the Brompton Hospital for Consumption, and £500 to the Maidenhead Cottage Hospital.

The position of Lincoln is not enviable in regard to its water supply. If the present resources are bad beyond remedy it would seem that there is nothing satisfactory to fall upon, and that a year or two at least must elapse before a reliable supply can be available.

Twenty-six ambulances for horses will be provided in the metropolis by Our Dumb Friends' League, which has established a special fund for the purpose. Four of these ambulances have been at work during the past few months. Lady Malcolm of Poltalloch has presented one by Messrs. Dennis, which can be summoned by telephone. The address of the League is 118 Victoria Street, S.W.

At the last meeting of the Board of Management of the Manchester Royal Infirmary, a proposal was considered to erect a temporary hospital during the building of the new infirmary, so that the site of the old building might be available and rendered productive. Mr. Bell, who put forward the proposal, contended that a considerable saving might be effected by its adoption. His method of calculating the financial gain was challenged and the motion was lost by a large majority.

In connection with the very pronounced epidemic of cerebro-spinal meningitis which has been present in New York ever since the beginning of the present year, the Health Department is organising a searching investigation which will include a house-to-house visitation. One hundred and fifty inspectors will be employed, and a health census will be taken. A similar epidemic is also prevalent in Silesia in Germany at the present time, and is regarded with the same serious attention as in America.

The recently published report by Dr. Gordon on the ventilation of the debating chamber of the House of Commons is a document which might be profitably studied by hospital authorities, especially in the light of the very marked differences of opinion which exist as to the best way of ventilating wards. Dr. Gordon found that the air of the debating chamber was altogether too dry, and in addition he observed that the upward ventilation in use is incapable of removing the particulate contaminating matter from the atmosphere. The effect of the ventilation at the time the experiments were made was, Dr. Gordon states, "merely to permit distribution of such material over a larger area in the Chamber than had been the case in a previous experiment when the ventilation was off."

